# Knowledge, Attitudes, and Practices of Occupational Therapists in Promoting Oral Health: A Protocol for Mixed-Methods Systematic Review

**DOI:** 10.3390/healthcare13040416

**Published:** 2025-02-14

**Authors:** Ming Nam Tse, Kristy Coxon, Navira Chandio, Shruti Nair, Ajesh George, Rosalind Bye, Grace Wong, Carol Tran, Maria O’Reilly, Kanchana Ekanayake, Amit Arora

**Affiliations:** 1 School of Health Sciences, Western Sydney University, Penrith, NSW 2751, Australia; 20437477@student.westernsydney.edu.au (M.N.T.); k.coxon@westernsydney.edu.au (K.C.); 18154463@student.westernsydney.edu.au (N.C.); shrutinair510@gmail.com (S.N.); r.bye@westernsydney.edu.au (R.B.); 2 Health Equity across Lifespan Laboratory, Campbelltown, NSW 2560, Australia; 3 Translational Health Research Institute, Western Sydney University, Campbelltown, NSW 2560, Australia; 4 Australian Centre for Integration of Oral Health (ACIOH), School of Nursing & Midwifery, Western Sydney University, Liverpool, NSW 1871, Australia; a.george@westernsydney.edu.au; 5 Sydney Dental School, Faculty of Medicine and Health, The University of Sydney, Westmead, NSW 2145, Australia; grace.wong4@health.nsw.gov.au; 6 School of Nursing, University of Wollongong, Wollongong, NSW 2522, Australia; 7 Ingham Institute for Applied Medical Research, Liverpool, NSW 1871, Australia; 8 Oral Health Services, Primary and Community Health, Northern Sydney Local Health District, NSW Health, North Ryde, NSW 2113, Australia; 9 School of Nursing and Midwifery, Western Sydney University, Penrith, NSW 2751, Australia; 10 Office of the Chief Dental Officer, Queensland Health, Brisbane, QLD 4000, Australia; c.tran10@gmail.com; 11 School of Health Medical and Applied Sciences, Central Queensland University, Bundaberg, QLD 4670, Australia; m.oreilly@cqu.edu.au; 12 Faculty of Medicine and Health, The University of Sydney, Camperdown, NSW 2006, Australia; kanchana.ekanayake@sydney.edu.au; 13 Discipline of Child and Adolescent Health, Faculty of Medicine and Health, The Children’s Hospital at Westmead Clinical School, The University of Sydney, Westmead, NSW 2145, Australia; 14 Oral Health Services, Sydney Local Health District and Sydney Dental Hospital, NSW Health, Surry Hills, NSW 2010, Australia

**Keywords:** oral health, occupational therapists, oral health promotion, interprofessional oral health, systematic review

## Abstract

Background/Objectives: Poor oral health is a significant global public health concern that adversely affects an individuals’ overall health and general well-being. Occupational therapists are well-suited to promote oral health by supporting activities of daily living (ADLs), thereby improving clients’ oral health outcomes. However, there is limited evidence on the knowledge, attitudes, and practices (KAPs) of occupational therapists regarding oral health, as well as the barriers and facilitators they face in delivering oral healthcare. Methods: This paper outlines a protocol for a mixed-methods systematic review aimed at synthesizing the current evidence on the KAPs of occupational therapists related to oral health, as well as the barriers and facilitators they encounter in promoting it. The upcoming systematic review will follow the Joanna Briggs Institute (JBI) methodology for mixed-methods systematic reviews using a convergent integrated approach to synthesis and integration. The review will include quantitative, qualitative, and mixed-methods studies that report on KAP, barriers, and facilitators associated with occupational therapists’ involvement in oral healthcare. A comprehensive search will be conducted across multiple databases, including MEDLINE (Ovid), CINAHL (EBSCO), SCOPUS, EMBASE (Ovid), and OTseeker to identify relevant studies. Two reviewers will independently screen the studies for eligibility, assess their methodological quality, and extract key data for synthesis. The protocol adheres to the Preferred Reporting Items for Systematic Review and Meta-Analysis Protocols (PRISMA-P) guidelines and is registered with PROSPERO (CRD42024522136). Results: The findings from the planned systematic review are expected to provide valuable insights into the role of occupational therapists in promoting oral health, addressing barriers and facilitators, and shaping policies, training programs, and clinical practices. Conclusions: Ultimately, these findings aim to enhance the integration of oral health into occupational therapy and improve client outcomes.

## 1. Introduction

Oral diseases are among the most prevalent non-communicable diseases, affecting 3.5 billion people globally in 2019 and imposing substantial health and economic burdens while significantly diminishing the oral health-related quality of life of those affected [1,2,3]. Common conditions, such as dental caries, periodontal disease, edentulism, and oral cancers, disproportionately impact vulnerable populations, particularly in low- and middle-income countries, as well as marginalized communities in high-income nations [1,4]. In high-income countries, barriers such as the lack of dental insurance, high out-of-pocket costs, and geographical disparities in service availability limit access to care, particularly for uninsured individuals and those in remote areas [5,6,7,8,9]. Similarly, in low- and middle-income countries, disadvantaged groups, such as those with lower socio-economic status, rural populations, and individuals with specialized health needs, face significant challenges in accessing oral healthcare [4]. Globally, three out of four people affected by oral diseases live in middle-income countries, exacerbating healthcare inequities and disproportionately affecting vulnerable populations such as racial and ethnic minorities, Indigenous communities, migrant populations, and rural residents experiencing poor oral health outcomes [5,6,7,8,9]. The high economic burden of oral diseases is evidenced by an expenditure exceeding 380 billion US dollars annually, representing approximately 4.8% of global direct health expenditures, with people of low socio-economic status carrying a higher burden of oral diseases throughout the life course. The extent of these disparities is often influenced by the structure of national health systems, with countries relying heavily on private dental care facing higher inequities compared to those offering universal dental services [7,10].

The rising global burden of oral diseases presents a major challenge for healthcare systems, particularly in regions with limited access to dental care and preventive strategies [4]. This challenge is amplified by the strong connection between oral health and systemic conditions, such as diabetes, cardiovascular diseases, and chronic kidney disease, underscoring the critical role oral health plays in overall well-being [11]. Untreated oral diseases can exacerbate glycemic control in people with diabetes and are associated with an increased risk of stroke and heart disease [12]. This interconnection underscores the importance of integrating oral health into general healthcare practices.

Access to quality oral healthcare remains a challenge for many people, particularly due to the high cost of private dental services, long waiting times in public healthcare systems, and a shortage of dental professionals in rural and remote areas [7,13]. Limited oral health literacy and treatment costs further perpetuate disparities, delaying necessary interventions and worsening oral conditions [14,15]. Addressing these delays is crucial to mitigating preventable oral conditions and promoting timely care across vulnerable populations [16,17].

In response to these challenges, international efforts have increasingly focused on engaging non-dental healthcare professionals to promote oral health, raise awareness, and provide basic oral care [18]. Nurses, midwives, and allied health practitioners have been identified as key players in addressing oral health due to their ongoing interaction with patients [19,20]. The World Health Organization’s (WHO) Global Oral Health Strategy (2024–2030) emphasizes the importance of interdisciplinary approaches to improving access to oral health services, reducing health inequities, and integrating oral health into universal health coverage (UHC) frameworks [18,21,22]. National oral health strategies in countries such as Australia, the United States, and Canada align with these goals by promoting interdisciplinary collaboration to enhance access to care [23,24,25]. Policy recommendations and targeted training programs are essential to equip non-dental healthcare professionals with the skills and knowledge needed to effectively address oral health disparities and improve access to care [18]. Existing collaborations between occupational therapists and dental professionals have demonstrated the benefits of interdisciplinary approaches, particularly in improving access for individuals with sensory and developmental challenges, such as autism spectrum disorders, and addressing oral health promotion in children through interprofessional education initiatives [17,26]. Similarly, the American Occupational Therapy Association (AOTA) highlights the need for integrating oral health into occupational therapy practice to address oral health disparities effectively [27].

Occupational therapists with their expertise in activities of daily living (ADLs), are well-equipped to promote oral hygiene as a fundamental aspect of personal care. Their role in self-care education enables them to assist populations facing barriers to oral care, such as older adults, individuals with disabilities, and those recovering from illness or injury [27,28,29]. While they are not expected to provide direct oral health services, additional training in oral care practices could enhance their ability to support clients effectively. Integrating oral care into occupational therapy practice can help improve access to oral health support, particularly for vulnerable populations who face systemic barriers to care. This approach addresses the broader need for interdisciplinary collaboration rather than acting as a replacement for dental professionals.

Despite their potential contribution to oral health promotion, systematic research on occupational therapists’ knowledge, attitudes, and practices (KAPs) related to oral health remains limited. There is also insufficient evidence on the barriers and facilitators they face in delivering oral healthcare. This paper presents a protocol for our mixed-methods systematic review aimed at synthesizing existing evidence on the KAPs, as well as the barriers and facilitators occupational therapists encounter in promoting oral health among clients. The objectives of the proposed review are two-fold:

1. To assess occupational therapists’ current KAP in promoting oral health
2. To identify the barriers and facilitators occupational therapists encounter when integrating oral health into their professional practice

This review aims to provide evidence to inform supplemental education and training initiatives that effectively support oral healthcare within the scope of practice for occupational therapists. Additionally, it will offer evidence-based recommendations for policy development and targeted training programs that strengthen interdisciplinary collaboration in oral healthcare delivery.

## 2. Methods

### 2.1. Protocol and Registration

This paper presents a protocol outlining the planned methodology for a mixed-methods systematic review, which will be conducted in accordance with the Joanna Briggs Institute (JBI) methodology for mixed-methods systematic reviews [30]. The planned systematic review will strictly adhere to this protocol and follow the PRISMA guidelines to ensure compliance with recognized standards for systematic reviews [31]. Additionally, this protocol has been developed in line with the PRISMA guidelines and reported following the Preferred Reporting Items for Systematic Review and Meta-Analyses Protocols (PRISMA-P) statement (see Appendix A. PRISMA-P checklist) [32,33], ensuring a structured and transparent approach. This protocol has been registered with PROSPERO (CRD42024522136).

### 2.2. Review Question

This protocol defines two research questions for the planned mixed-methods systematic review using the Population, Concept, and Context (PCC) framework [34]:

1. What are the current KAPs of occupational therapists regarding oral healthcare?
2. What are the barriers and facilitators occupational therapists face when delivering oral healthcare to clients.

### 2.3. Eligibility Criteria

The eligibility criteria for the upcoming systematic review are as follows:

#### 2.3.1. Inclusion Criteria

Study Design: Quantitative, qualitative, and mixed-methods studies will be included in the systematic review. Only studies published in English language in peer-reviewed journals will be included, with no restrictions on publication date.

Population: Occupational therapists working in community health centers, hospitals, disability services, aged care services, schools, and private practices.

Concept: The systematic review will focus on two key conceptual areas:

(a) KAP: This concept will explore occupational therapists’ level of knowledge about oral health, attitudes towards incorporating oral health into their practice, and how these factors will translate into their daily professional practices. The purpose of this review is to assess how occupational therapists understand and value oral health, and how this understanding and valuation affects their practical approach to client care.
(b) Barriers and Facilitators: The review will examine the various barriers and enablers influencing the integration of oral healthcare into occupational therapy. It will specifically focus on how these factors influence occupational therapists’ ability to support clients’ oral health within their professional scope. Barriers to promoting oral health may include systemic challenges, such as limited oral health training, policy restrictions that limit interdisciplinary collaboration with dental professionals, resource constraints, unclear guidelines on incorporating oral health into occupational therapy practice, and other factors that hinder effective delivery of oral healthcare. Conversely, this review will also explore facilitators that support the integration of oral healthcare, such as policies that emphasize the significance of oral health in overall well-being, training programs designed to equip occupational therapists with essential skills in promoting oral hygiene, opportunities for interdisciplinary collaboration with oral health professionals, and effective communication strategies that empower occupational therapists to educate clients on maintaining oral hygiene routines.

Context: The systematic review will examine the broader domain of oral health, including topics such as dental health, oral hygiene practices, dental care services, prevention and management of dental caries, and factors influencing dental visits. It will also address related aspects, such as tooth and mouth diseases, gum diseases, and the maintenance of oral health. This comprehensive scope will provide insights into how occupational therapy intersects with various dimensions of oral healthcare.

#### 2.3.2. Exclusion Criteria

Studies with the following criteria will be excluded from the review:

- Conference abstracts and reports, editorial, discussions, study protocols, commentaries, response articles and systematic reviews, and meta-analyses
- Articles published in other languages.

### 2.4. Information Sources

The following electronic databases will be searched without any restriction on publication date, type, or region: MEDLINE (Ovid), CINAHL (EBSCO), SCOPUS, EMBASE (Ovid), and OTseeker. These databases have been selected due to their relevance to the field of allied health and occupational therapy. Only studies published in peer-reviewed English-language journals will be included. Gray literature will not be considered for this review.

### 2.5. Search Strategy

A preliminary search of several databases was conducted to determine if similar systematic reviews currently exist. The search strategy of this study will be developed by following the PCC framework [34], which will be used to formulate the search terms:

Population: Occupational therapists.
Concept: KAP, facilitator, and barrier.
Context: Oral health.

A search strategy has been developed by creating a logic grid (See Appendix B) and developed for MEDLINE (Ovid) with assistance from a health science librarian (See Appendix C) [35]. A comprehensive search implementing Boolean terms, keywords, MeSH terms, and wildcards was conducted to locate and examine potential studies that are relevant to the PCC components and the review question [36]. A manual search of the relevant studies’ reference lists will be conducted to identify additional applicable studies and articles. The MEDLINE (Ovid) search strategy will be tailored to suit the different subject headings and syntaxes of the other databases. Study authors will be contacted if full-text articles are not available. If there is no response after two attempts, we will exclude the article.

### 2.6. Study Selection Process

Studies identified through databases and manual search will be managed using Endnote 21 reference management software [37]. Studies will be imported to Covidence software, and duplicates will be removed. Two reviewers (M.N.T. and A.A.) will undertake title and abstract screening based on a priori selection criteria. Studies that meet the inclusion criteria or if the study eligibility is unclear, will undergo a full-text review.

The full text of all eligible studies will be independently assessed by two reviewers (M.N.T. and A.A.) to determine final inclusion in the systematic review. Study authors will be contacted if any further information is required. If there are any disagreements regarding study inclusion or exclusion, a third reviewer (N.C. or K.C.) will be approached to make the final decision. If any studies are excluded after a full-text review, the reasons for excluding studies will be documented, and the results will be presented in a PRISMA flow diagram.

### 2.7. Quality Assessment

The methodological quality of all included studies will be assessed by two reviewers (M.N.T. and N.C.) using JBI, North Adelaide, South Australia, critical appraisal tools [30]. If there are any disagreements regarding the study’s methodological quality, a third team member (A.A. or K.C.) will be approached to reach a consensus. The methodological quality of all included studies will be presented as a table and will be accompanied by a narrative summary.

### 2.8. Data Extraction

A standard data extraction form will be created to collect information for the systematic review. The extracted data will be put into a word document with the listed headings to include specific details about the study, including reference, study aim, setting and design, participants, and outcomes and limitations. Prior to the full-scale data extraction, a pilot test on three studies will be conducted to ensure the tool’s reliability and consistency across reviews. Two reviewer authors (M.N.T. and A.A./N.C./K.C.) will extract data independently. Other team members will be consulted to resolve any discrepancies identified during the data extraction as needed.

The data extraction will include the following key elements:

1. Study reference—citation details, including authors, title, and publication year.
2. Study aim—the primary objectives or hypotheses of the study.
3. Study setting—study location, including city and country.
4. Study design—quantitative (e.g., cross-sectional, cohort, case–control, intervention studies) or qualitative studies, or mixed-methods studies.
5. Participants—occupational therapists working in various clinical settings (e.g., hospitals, community health services, long-term care facilities, and private practices).
6. Outcomes measured–KAP, barriers, and facilitators related to the integration of oral healthcare into occupational therapy practice.
7. Study limitations—any limitations or biases noted by the study authors.

### 2.9. Data Synthesis

Included studies will be categorized according to study design. For qualitative data, a thematic synthesis approach will be utilized [38,39]. Two authors will independently code the qualitative findings, identifying themes and subthemes related to the KAPs of occupational therapists. The coding process will encompass study demographics, design, and the specific qualitative outcomes related to barriers, facilitators, and other factors impacting occupational therapists’ roles in oral healthcare. These descriptive themes will then be reviewed, with the authors collaborating to develop analytical themes and sub-themes, which will offer deeper insights into the relationship between the findings and the research objectives [38,40].

For categorical data, frequencies and percentages will be reported. For continuous data, mean and standard deviations (SDs) or median and interquartile range will be reported. If studies report similar outcomes, we will pool the results to provide an overall estimate. The results will be presented as pooled proportions, 95% confidence intervals, range, and pooled frequencies for each outcome. A Cochrane Q-test will be used to test for heterogeneity of proportions [41]. Random effects model of meta-analysis will be used due to anticipated heterogeneity in study results [42]. If data are available, a sub-group analysis will be performed (e.g., based on country or study setting). Sensitivity analyses will be performed on studies with a low risk of bias. If meta-analysis is not possible, the findings will be presented in a narrative format, complemented by figures and tables [42].

### 2.10. Meta-Biases

We will conduct a thorough evaluation of publication bias and selective reporting within studies to assess for potential meta-biases in this review. Publication bias will be assessed through a funnel plot if enough studies (at least 10) report comparable outcomes. Additionally, an Egger’s test will be applied to quantitatively evaluate asymmetry in the funnel plot, which may suggest publication bias. Selective reporting will be checked by comparing the study protocols, if available, to the reported outcomes in each study. Any discrepancies found between the protocol outcomes and reported outcomes will be noted, as they may indicate selective reporting within studies [43].

## 3. Discussion

This protocol establishes a structured framework to investigate the KAPs of occupational therapists regarding oral healthcare, alongside the barriers and facilitators influencing its integration into clinical practice. The planned systematic review is designed to address critical gaps in knowledge and practice by analyzing systemic and operational challenges encountered by occupational therapists. By doing so, this review aims to generate evidence-based recommendations that inform policy development and guide the creation of targeted training programs, ultimately enhancing the integration of oral healthcare within occupational therapy practice.

A notable strength anticipated from the planned systematic review, as outlined in this protocol, is the use of its rigorous mixed-methods design, incorporating both quantitative and qualitative data. This approach ensures a comprehensive understanding of the topic by not only quantifying trends in the KAPs of occupational therapists but also offering thematic insights into contextual factors and lived experiences that influence their practices. Adherence to the Joanna Briggs Institute (JBI) methodology further ensures methodological rigor and consistency, enhancing the reliability and validity of the findings. Additionally, this protocol employs a well-structured Population, Concept, and Context (PCC) framework for systematic searching, allowing for a focused yet inclusive capture of evidence. These methodological strengths are expected to yield actionable insights, particularly in regions with limited access to dental professionals, where occupational therapists could play a vital role in bridging gaps in oral healthcare.

Despite these strengths, the planned review may encounter certain limitations. The exclusion of studies published in languages other than English may restrict the global applicability of the findings by overlooking relevant research from non-English-speaking regions. Furthermore, limiting the review to peer-reviewed literature could introduce a publication bias by excluding valuable insights from gray literature. Recognizing these potential limitations, this review emphasizes the importance of transparent documentation and critical interpretation of findings to mitigate biases and provide a balanced synthesis.

Ultimately, this systematic review is expected to offer valuable insights into the role of occupational therapists in promoting oral health, highlighting both challenges and facilitators to their involvement. By addressing systemic barriers and leveraging facilitators such as interdisciplinary collaboration and targeted training, the findings aim to inform actionable recommendations for policy development and practice. This contribution is anticipated to advance the integration of oral healthcare within occupational therapy and improve outcomes for clients, particularly those in vulnerable populations.

## 4. Conclusions

The systematic review outlined in this protocol aims to synthesize existing evidence on the KAPs of occupational therapists regarding oral healthcare, while identifying the barriers and facilitators influencing its integration into practice. Following the rigorous JBI methodology and PRISMA-P guidelines, this review will provide a comprehensive analysis of the topic using diverse data sources. The findings from the review are anticipated to guide the development of targeted training programs and inform policy initiatives by addressing critical gaps in knowledge and practice. By empowering occupational therapists to collaborate across disciplines and implement tailored interventions, this review seeks to enhance their role in promoting oral health, particularly for vulnerable populations, ultimately advancing the integration of oral healthcare into occupational therapy and contributing to improved client outcomes and reduced health inequities.

## Data Availability

Not applicable.

## Appendix A. PRISMA-P 2015 Checklist [32]

**Section/Topic**	**#**	**Checklist Item**	**Information** **Reported**		**Line** **Number(s)**
			**Yes**	**No**	
**ADMINISTRATIVE INFORMATION**					
**Title**					
Identification	1a	Identify the report as a protocol of a systematic review			2–4
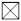
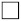
Update	1b	If the protocol is for an update of a previous systematic review, identify as such			NA
**Registration**	2	If registered, provide the name of the registry (e.g., PROSPERO) and registration number in the Abstract			43
**Authors**					
Contact	3a	Provide name, institutional affiliation, and e-mail address of all protocol authors; provide physical mailing address of corresponding author			5–25
Contributions	3b	Describe contributions of protocol authors and identify the guarantor of the review			338–340
**Amendments**	4	If the protocol represents an amendment of a previously completed or published protocol, identify as such and list changes; otherwise, state plan for documenting important protocol amendments			NA
**Support**					
Sources	5a	Indicate sources of financial or other support for the review			341
Sponsor	5b	Provide name for the review funder and/or sponsor			NA
Role of sponsor/funder	5c	Describe roles of funder(s), sponsor(s), and/or institution(s), if any, in developing the protocol			NA
**INTRODUCTION**					
**Rationale**	6	Describe the rationale for the review in the context of what is already known			51–133
**Objectives**	7	Provide an explicit statement of the question(s) the review will address with reference to participants, interventions, comparators, and outcomes (PICO)			125–128
**METHODS**					
**Eligibility criteria**	8	Specify the study characteristics (e.g., PICO, study design, setting, time frame) and report characteristics (e.g., years considered, language, publication status) to be used as criteria for eligibility for the review			153–194
**Information sources**	9	Describe all intended information sources (e.g., electronic databases, contact with study authors, trial registers, or other gray literature sources) with planned dates of coverage			195–201
**Search strategy**	10	Present draft of search strategy to be used for at least one electronic database, including planned limits, such that it could be repeated			202–218
***STUDY RECORDS***					
Data management	11a	Describe the mechanism(s) that will be used to manage records and data throughout the review			220–222
Selection process	11b	State the process that will be used for selecting studies (e.g., two independent reviewers) through each phase of the review (i.e., screening, eligibility, and inclusion in meta-analysis)			219–231
Data collection process	11c	Describe planned method of extracting data from reports (e.g., piloting forms, performed independently, in duplicate), any processes for obtaining and confirming data from investigators			238–257
**Data items**	12	List and define all variables for which data will be sought (e.g., PICO items, funding sources), any pre-planned data assumptions and simplifications			238–257
**Outcomes and prioritization**	13	List and define all outcomes for which data will be sought, including prioritization of main and additional outcomes, with rationale			255–256
**Risk of bias in individual studies**	14	Describe anticipated methods for assessing risk of bias of individual studies, including whether this will be conducted at the outcome or study level, or both; state how this information will be used in data synthesis			232–237
***DATA***					
**Synthesis**	15a	Describe criteria under which study data will be quantitatively synthesized			268–277
	15b	If data are appropriate for quantitative synthesis, describe planned summary measures, methods of handling data, and methods of combining data from studies, including any planned exploration of consistency (e.g., *I*^2^, Kendall’s tau)			268–277
	15c	Describe any proposed additional analyses (e.g., sensitivity or subgroup analyses, meta-regression)			275–277
	15d	If quantitative synthesis is not appropriate, describe the type of summary planned			276–277
**Meta-bias(es)**	16	Specify any planned assessment of meta-bias(es) (e.g., publication bias across studies, selective reporting within studies)			279–286
**Confidence in cumulative evidence**	17	Describe how the strength of the body of evidence will be assessed (e.g., GRADE)			NA
		This box is empty. This box has been selected. NA = Not available.			

## Appendix B. Population, Concept and Context (PCC) Logic Grid [35]

	**Key Word**	**Synonyms**	**Index Search (Keywords)**
Population	Occupational therapist	- Occupational therapy - Allied health	Occupational therap*
Concept	Health knowledge, attitude and practice, Barrier and facilitator	- Health knowledge - Practice - Attitude - Challenge - Enabler - Obstacle - Procedure	(knowledge or practice* or health practice* or procedure* or attitude* or barrier* or challenge* or facilitator* or enabler* or obstacle*).mp.
Context	Oral health	- Dental health - Oral hygiene - Dental care - Dental caries - Dental visit - Teeth - Tooth - Mouth disease - Gum disease - Toothbrushing	((Oral or dental or teeth or tooth or gum) adj3 (health or hygiene or car* or treatment* or visit* or diseas*)).mp.

## Appendix C. MEDLINE (Ovid) Search Strategy

**#**	**Query**	**Results from 9 October 2024**
1	exp Oral Health/ or exp Oral Hygiene/ or exp Dental Care/ or exp Dental Care for Children/ or exp Dental Care for Disabled/ or exp Gingivitis/ or exp Periodontal Diseases/ or exp Chronic Periodontitis/ or exp Dental Plaque/ or exp Periodontitis/ or exp Tooth Diseases/ or exp Dental Caries/ or exp Mouth Diseases/ or exp Mouth Neoplasms/ or exp Tooth Injuries/ or exp Dentition, Mixed/ or exp Dentition/ or exp Dentition, Permanent/ or exp Toothache/ or exp Toothbrushing/ or exp Education, Dental/ or Dental Care for Aged/ or Denture Stomatitis/	644,253
2	(oral health* or dental health* or oral disease* or dental disease* or dental caries* or tooth decay* or oral trauma* or gum disease* or periodont* or gingiv* or oral hygiene* or mouth diseas* or dental car* or mouth tumo?r* or Mouth Neoplasm* or mouth cancer* or Oral cancer* or Oral traum* or dental visit* or tooth loss* or dental status* or dental pain* or dentition* or teeth* or tooth diseas* or dental care* or Tooth Injur* or toothache* or toothache* or toothbrushing* or dental educat* or tooth diseas* or teeth diseas* or denture clean* or Dental Plaque* or Denture Stomatitis).mp.	481,729
3	((Oral or dental or gum) adj2 (health* or hygiene* or car* or treatment* or visit* or diseas* or plaque)).tw,kw.	152,418
4	1 or 2 or 3	814,847
5	Occupational Therapy/ or Occupational Therapists/ or patient care team/ or “activities of daily living”/	158,595
6	(occupational therap* or ergotherap* or OT therap* or “activities of daily living”).mp.	115,175
7	(Allied health* or Multidisciplinary team* or Multi-disciplinary team* or patient care team*).tw,kw.	46,077
8	5 or 6 or 7	222,913
9	Health Knowledge, Attitudes, Practice/ or Health behavior/ or Attitude to health/ or health knowledge, attitudes, practice/ or capacity building/ or health communication/ or “attitude of health personnel”/	373,859
10	(Health behavio?r or “Attitude to health” or health belief* or health practice* or health literac* or knowledge or attitude* or practice* or procedure* or interdisciplinary or multidisciplinary or interprofessional*).mp.	4,441,036
11	(barrier* or impediment* or challenge* or hindrance* or obstacle* or hurdle* or obstruction* or deterrent* or facilitator*).mp.	1,821,644
12	9 or 10 or 11	5,843,677
13	4 and 8 and 12	3118
	* = Wildcard symbol that broadens a search by finding words that start with the same letters.	

## References

[1] World Health Organization (2022). Global Oral Health Status Report: Towards Universal Health Coverage for Oral Health by 2030.

[2] Sischo L., Broder H.L. (2011). Oral health-related quality of life: What, why, how, and future implications. J. Dent. Res..

[3] Spanemberg J.C., Cardoso J.A., Slob E.M.G.B., López-López J. (2019). Quality of life related to oral health and its impact in adults. J. Stomatol. Oral Maxillofac. Surg..

[4] Peres M.A., Macpherson L.M., Weyant R.J., Daly B., Venturelli R., Mathur M.R., Listl S., Celeste R.K., Guarnizo-Herreño C.C., Kearns C. (2019). Oral diseases: A global public health challenge. Lancet.

[5] World Health Organization Oral Health. https://www.who.int/news-room/fact-sheets/detail/oral-health.

[6] Bernabé E., Masood M., Vujicic M. (2017). The impact of out-of-pocket payments for dental care on household finances in low and middle income countries. BMC Public Health.

[7] Northridge M.E., Kumar A., Kaur R. (2020). Disparities in Access to Oral Health Care. Annu. Rev. Public Health.

[8] Australian Institute of Health Welfare Oral Health and Dental Care in Australia. https://www.aihw.gov.au/reports/dental-oral-health/oral-health-and-dental-care-in-australia.

[9] Khan A.J., Md Sabri B.A., Ahmad M.S. (2022). Factors affecting provision of oral health care for people with special health care needs: A systematic review. Saudi Dent. J..

[10] Chari M., Ravaghi V., Sabbah W., Gomaa N., Singhal S., Quiñonez C. (2022). Oral health inequality in Canada, the United States and United Kingdom. PLoS ONE.

[11] Kotronia E., Brown H., Papacosta A.O., Lennon L.T., Weyant R.J., Whincup P.H., Wannamethee S.G., Ramsay S.E. (2021). Oral health and all-cause, cardiovascular disease, and respiratory mortality in older people in the UK and USA. Sci. Rep..

[12] King S., Chow C.K., Eberhard J. (2022). Oral health and cardiometabolic disease: Understanding the relationship. Intern. Med. J..

[13] Crocombe L.A., Chrisopoulos S., Kapellas K., Brennan D., Luzzi L., Khan S. (2022). Access to dental care barriers and poor clinical oral health in Australian regional populations. Aust. Dent. J..

[14] Barnett T., Hoang H., Stuart J., Crocombe L. (2017). The relationship of primary care providers to dental practitioners in rural and remote Australia. BMC Health Serv. Res..

[15] Levy B.B., Goodman J., Eskander A. (2023). Oral healthcare disparities in Canada: Filling in the gaps. Can. J. Public Health.

[16] da Rosa S.V., Moysés S.J., Theis L.C., Soares R.C., Moysés S.T., Werneck R.I., Rocha J.S. (2020). Barriers in Access to Dental Services Hindering the Treatment of People with Disabilities: A Systematic Review. Int. J. Dent..

[17] Como D.H., Stein Duker L.I., Polido J.C., Cermak S.A. (2021). Oral Health and Autism Spectrum Disorders: A Unique Collaboration between Dentistry and Occupational Therapy. Int. J. Environ. Res. Public Health.

[18] World Health Organization (2024). Global strategy and action plan on oral health 2023–2030. Global Strategy and Action Plan on Oral Health 2023–2030.

[19] Fisher J., Berman R., Buse K., Doll B., Glick M., Metzl J., Touger-Decker R. (2023). Achieving Oral Health for All through Public Health Approaches, Interprofessional, and Transdisciplinary Education. NAM Perspect..

[20] Prasad M., Manjunath C., Murthy A.K., Sampath A., Jaiswal S., Mohapatra A. (2019). Integration of oral health into primary health care: A systematic review. J. Fam. Med. Prim. Care.

[21] World Health Organization (2016). Framework on Integrated, People-Centred Health Services.

[22] Faisal M.R., Mishu M., Jahangir F., Younes S., Dogar O., Siddiqi K., Torgerson D.J. (2022). The effectiveness of behaviour change interventions delivered by non-dental health workers in promoting children's oral health: A systematic review and meta-analysis. PLoS ONE.

[23] COAG (Council of Australian Governments) Health Council (2015). Healthy Mouths, Healthy Lives: Australia’s National Oral Health Plan 2015–2024.

[24] Office of Disease Prevention and Health Promotion, U.S. Department of Health and Human Services Healthy People 2030 Framework. https://odphp.health.gov/healthypeople/about/healthy-people-2030-framework.

[25] Rock L.D., Allison P.J. (2024). A milestone for the oral health community: Canada’s first National Oral Health Research Strategy (2024–2030). Can. J. Dent. Hyg..

[26] Cermak S.A., Stein Duker L.I., Williams M.E., Dawson M.E., Lane C.J., Polido J.C. (2015). Sensory adapted dental environments to enhance oral care for children with autism spectrum disorders: A randomized controlled pilot study. J. Autism Dev. Disord..

[27] American Occupational Therapy Association (2020). Occupational Therapy Practice Framework: Domain et Process.

[28] Holmberg V., Ringsberg K.C. (2014). Occupational therapists as contributors to health promotion. Scand. J. Occup. Ther..

[29] Junnarkar V.S., Tong H.J., Hanna K.M.B., Aishworiya R., Duggal M. (2022). Occupational and speech therapists’ perceptions of their role in dental care for children with autism spectrum disorder: A qualitative exploration. Int. J. Paediatr. Dent..

[30] Lizarondo L.S.C., Carrier J., Godfrey C., Rieger K., Salmond S., Apostolo J., Kirkpatrick P., Loveday H., Aromataris E., Lockwood C., Porritt K., Pilla B., Jordan Z. (2020). Chapter 8: Mixed methods systematic reviews. JBI Manual for Evidence Synthesis.

[31] Page M.J., McKenzie J.E., Bossuyt P.M., Boutron I., Hoffmann T.C., Mulrow C.D., Shamseer L., Tetzlaff J.M., Akl E.A., Brennan S.E. (2021). The PRISMA 2020 statement: An updated guideline for reporting systematic reviews. Bmj.

[32] Moher D., Shamseer L., Clarke M., Ghersi D., Liberati A., Petticrew M., Shekelle P., Stewart L.A., Group P.-P. (2015). Preferred reporting items for systematic review and meta-analysis protocols (PRISMA-P) 2015 statement. Syst. Rev..

[33] Shamseer L., Moher D., Clarke M., Ghersi D., Liberati A., Petticrew M., Shekelle P., Stewart L.A. (2015). Preferred reporting items for systematic review and meta-analysis protocols (PRISMA-P) 2015: Elaboration and explanation. Bmj.

[34] Pollock D., Peters M.D., Khalil H., McInerney P., Alexander L., Tricco A.C., Evans C., de Moraes É.B., Godfrey C.M., Pieper D. (2023). Recommendations for the extraction, analysis, and presentation of results in scoping reviews. JBI Evid. Synth..

[35] Aromataris E., Riitano D. (2014). Constructing a search strategy and searching for evidence. A guide to the literature search for a systematic review. Am. J. Nurs..

[36] Khan K., Kunz R., Kleijnen J., Antes G. (2011). Systematic Reviews to Support Evidence-Based Medicine.

[37] The EndNote Team (2013). EndNote.

[38] Braun V., Clarke V. (2006). Using thematic analysis in psychology. Qual. Res. Psychol..

[39] Quirkos Software (2017). Quirkos Version 1.4.1 [Computer Software].

[40] Attride-Stirling J. (2001). Thematic networks: An analytic tool for qualitative research. Qual. Res..

[41] Egger M.S.G., Altman D. (2001). Systematic Reviews in Health Care: Meta-Analysis in Context.

[42] Moola S., Munn Z., Sears K., Sfetcu R., Currie M., Lisy K., Tufanaru C., Qureshi R., Mattis P., Mu P. (2015). Conducting systematic reviews of association (etiology): The Joanna Briggs Institute’s approach. JBI Evid. Implement..

[43] Page M.J., Higgins J.P., Sterne J.A. (2019). Assessing risk of bias due to missing results in a synthesis. Cochrane Handb. Syst. Rev. Interv..

